# “Floating shoulder” injuries

**DOI:** 10.1186/s12245-016-0110-y

**Published:** 2016-03-09

**Authors:** Kenneth Heng

**Affiliations:** Emergency Department, Tan Tock Seng Hospital Singapore, 11 Jalan Tan Tock Seng, Singapore, 308433 Singapore

**Keywords:** Floating shoulder, Scapulothoracic dissociation, Neurovascular injury

## Abstract

“Floating shoulder” is a rare injury complex resulting from high-energy blunt force trauma to the shoulder, resulting in scapulothoracic dissociation. It is commonly associated with catastrophic neurovascular injury. Two cases of motorcyclists with floating shoulder injuries are described.

## Background

Two cases of high-energy shoulder injuries involving motorcyclists are described. “Floating shoulder” injury, or scapulothoracic dissociation, is rare [1–3], but this unique injury pattern should be recognized earlier as it has devastating neurovascular implications and its management often requires a multi-disciplinary approach.

## Case presentation

### Case 1

**Fig. 1:**
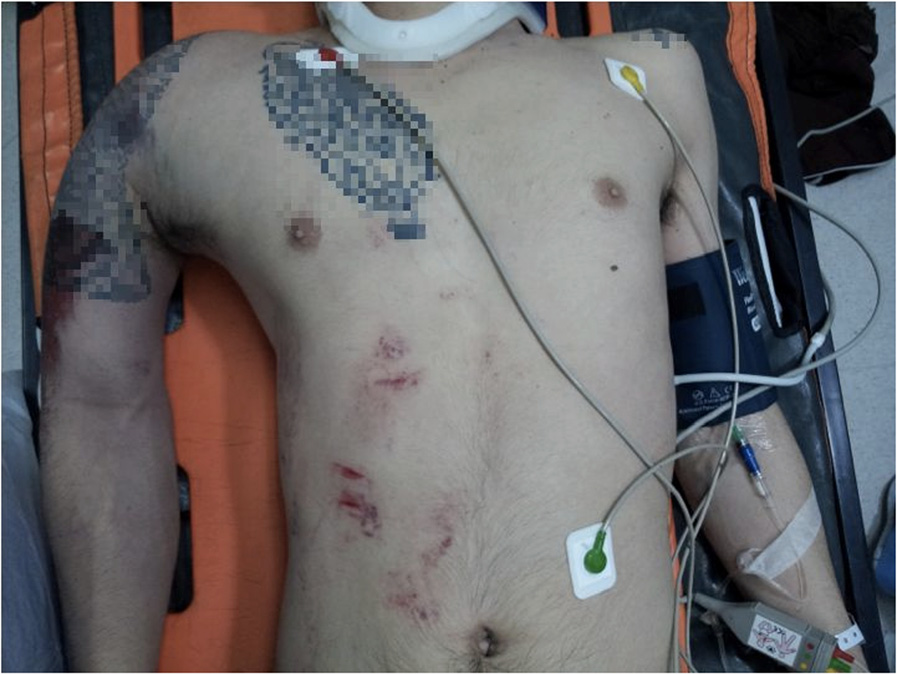
Right “floating shoulder.” Marked soft tissue swelling and deformity of the right shoulder and right chest wall

**Fig. 2:**
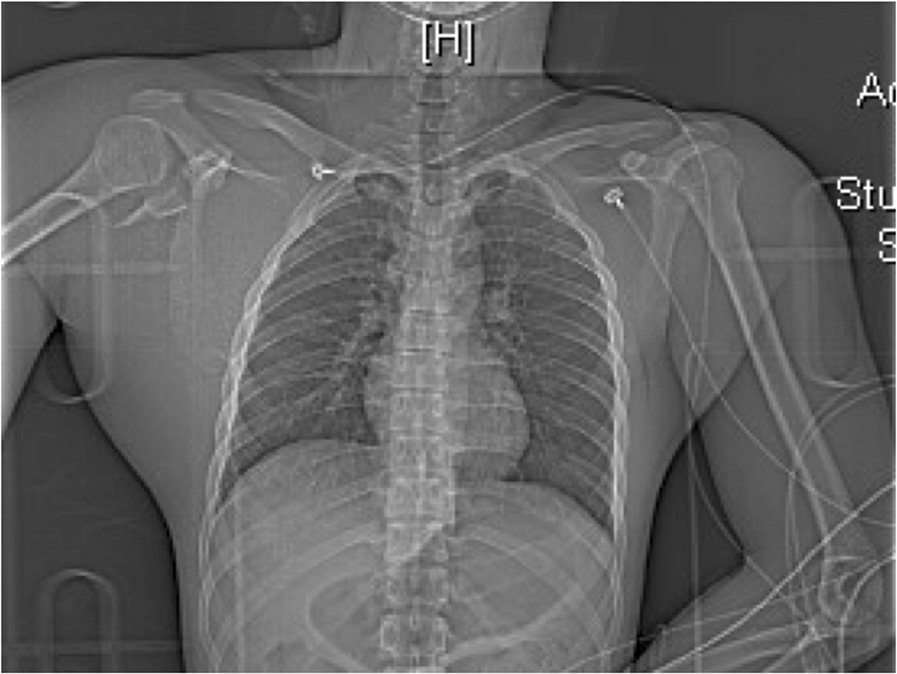
CT scout film. CT showing a fracture through the right scapular neck and marked soft tissue swelling of the right shoulder and chest wall

A 20-year-old male helmeted, alcohol-intoxicated motorcyclist lost control of his bike on the highway and fell. His pillion rider died at scene. On arrival at the emergency department, his blood pressure was 54/32, pulse rate 144/min, respiratory rate 16/min, and Glasgow Coma score 15. His main injuries were closed deformity of the right humerus, right chest wall, right shoulder (Fig. 1), and right iliac crest bruising. Point-of-care ultrasound showed no free intraperitoneal fluid. Pelvic radiography showed a fracture of the right iliac wing with no disruption of the pelvic ring. Scout computed tomography (CT) radiograph shows a fracture through the right scapular neck (Fig. 2) and marked soft tissue swelling of the right shoulder and chest wall. Apart from self-limited cancellous bone bleeding from the iliac wing fracture, pan-CT and pelvic angiography ruled out intrathoracic, abdominal, and pelvic source of bleeding. CT demonstrated right axillary artery disruption with massive chest wall hematoma, and the hypotension was attributed to this injury. The patient was resuscitated and underwent right axillo-brachial bypass graft with external fixation of the right humeral fracture. The median and ulnar nerves were avulsed proximally requiring repair. Unfortunately, his bypass graft occluded and he eventually required amputation of the arm at the shoulder.

### Case 2

**Fig. 3:**
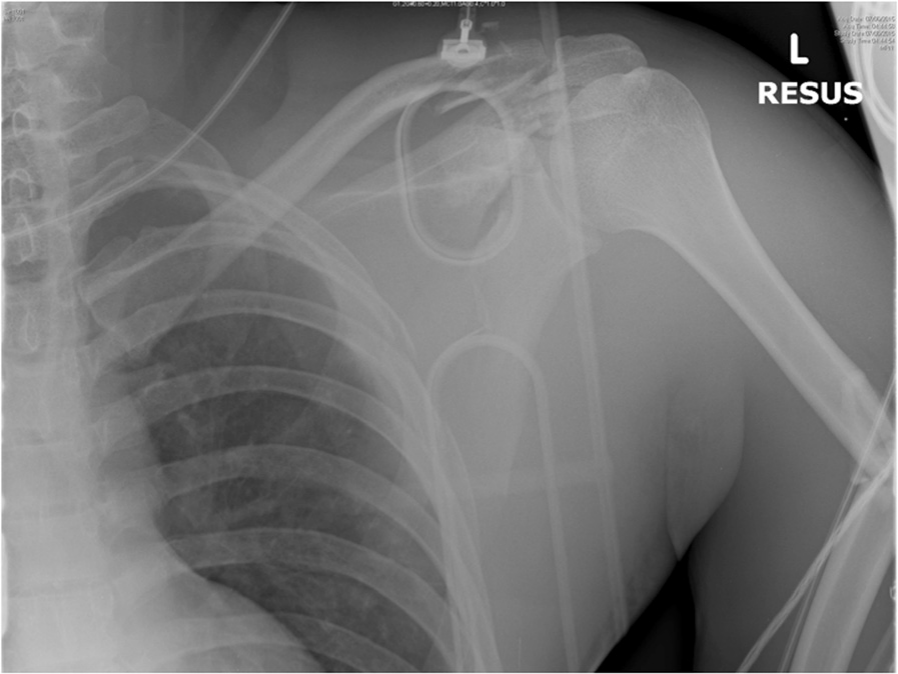
Plain X-ray of the left shoulder. Plain X-ray of the left shoulder showing fractures of the left scapula body, left humerus, left distal clavicle, left 3–8th ribs, and left chest wall soft tissue swelling

**Fig. 4:**
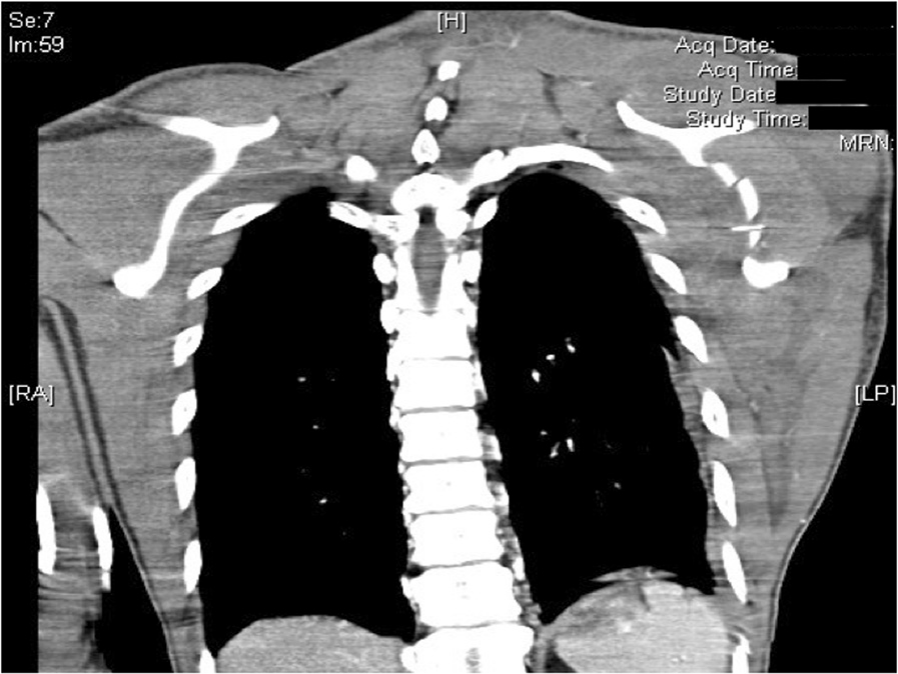
CT thorax coronal view. CT thorax showing comminuted fracture of the left scapula body and left chest wall soft tissue swelling

A 25-year-old motorcyclist traveling at 100 km per h was struck in the rear by a car. His vital parameters at scene were normal, and his main injury was to the left arm with no power of hand and elbow movement and diminished distal pulses. Plain and CT radiographs showed left scapula body fracture, a comminuted fracture of the left humerus, left distal clavicle fracture, left 3–8th rib fracture, and left chest wall soft tissue swelling (Figs. 3 and 4). He was in class 1 shock requiring fluid and blood products. Operative exploration showed a transected axillary artery. He underwent fixation of the left distal clavicle and humerus and endovascular stenting, which subsequently thrombosed. He subsequently successfully underwent a left axillary-brachial artery bypass graft. Left brachial plexus exploration showed post-ganglionic C5–C8 injury which required repair.

### Discussion

A floating shoulder represents a high-energy injury and is defined as a fracture of the scapular neck, ligament disruption, with or without a clavicular fracture [4]. If the patient is upright, the affected limb often hangs lower than the contralateral side. Due to its proximity, the axillary vessels and brachial plexus are commonly injured [5, 6]. If a patient with floating shoulder presents with hypotension, bleeding from concomitant injuries in the traditional areas “blood on the floor plus four more” (intrathoracic, intraperitoneal, retroperitoneal, pelvis/thigh) needs to be excluded. However, as shown in both cases, hemorrhage from the right axillary artery into the soft tissue of the axilla and chest wall alone can result in hypovolemic shock due to the large amount of potential space where blood may accumulate. In case 1, there was delayed surgical hemostasis as the team elected to first perform radiographic investigations to look for a pelvic or intra-abdominal source of bleeding. It is recommended that in similar floating shoulder cases where there is no hemothorax or widened mediastinum on the chest radiograph and pelvic radiograph does not show pelvic disruption and point-of-care ultrasound does not identify hemoperitoneum, urgent transfer to the operating theater for exploration and hemostasis of axillary artery is required, bypassing unnecessary CT scans.

## Conclusions

These two cases highlight the catastrophic neurovascular injuries that can occur with floating shoulder injuries, requiring prompt recognition, and the involvement of a multi-disciplinary team of orthopedic, trauma, and vascular surgeons to coordinate the management of this injury.

## Consent

Written informed consent was obtained from the patient for publication of this case report and any accompanying images. A copy of the written consent is available for review by the Editor-in-Chief of this journal.
